# Identification of TGFβ signaling as a regulator of interneuron neurogenesis in a human pluripotent stem cell model

**DOI:** 10.1042/NS20210020

**Published:** 2021-12-07

**Authors:** Maria Cruz Santos, Meng Li

**Affiliations:** 1 Neuroscience and Mental Health Research Institute, School of Medicine, Cardiff University, Cardiff CF24 4HQ, United Kingdom; 2 School of Biosciences, Cardiff University, Cardiff CF10 3AX, United Kingdom

**Keywords:** cell cycle, hESC, in vitro differentiation, interneurons, neurogenesis, TGFbeta

## Abstract

Cortical interneurons are GABAergic inhibitory cells that connect locally in the neocortex and play a pivotal role in shaping cortical network activities. Dysfunction of these cells is believed to lead to runaway excitation underlying seizure-based diseases, such as epilepsy, autism and schizophrenia. There is a growing interest in using cortical interneurons derived from human pluripotent stem cells for understanding their complex development and for modeling neuropsychiatric diseases. Here, we report the identification of a novel role of transforming growth factor β (TGFβ) signaling in modulating interneuron progenitor maintenance and neuronal differentiation. TGFβ signaling inhibition suppresses terminal differentiation of interneuron progenitors, while exogenous TGFβ3 accelerates the transition of progenitors into postmitotic neurons. We provide evidence that TGFb signaling exerts this function via regulating cell cycle length of the NKX2.1+ neural progenitors. Together, the present study represents a useful platform for studying human interneuron development and interneuron-associated neurological diseases with human pluripotent stem cells.

## Introduction

Cortical interneurons are cells that connect only with nearby neurons in the neocortex and express the inhibitory neurotransmitter γ-amino butyric acid (GABA). Despite these common features, interneurons display huge diversity in morphology, physiology and marker expression [1]. The different subtypes of interneurons shape the responses of glutamatergic projection neurons to incoming inputs, prevent hyperexcitability and are intimately involved in the timing and synchronization of cortical neuronal oscillations, thereby modulate cortical network activity with high spatiotemporal control [2]. Consequently, disruption of cortical GABAergic interneuron function, that may arise as a consequence of abnormal cortical development, has been linked to major neurological and psychiatric illnesses including epilepsy, autism, schizophrenia as well as dementia [1,3,4]. Interneurons that expresses somatostatin (SST) and parvalbumin (PV) appear to be preferentially affected in many of these disorders [4–6].

The SST and PV interneurons originate primarily from the medial ganglionic eminence (MGE), a structure that lies in the ventral-most aspect of the developing forebrain [7–11]. MGE neural progenitors are coded by the transcription factor NKX2.1, whose expression is vital for MGE-based interneurogenesis, whereby its knockout in mice reduced GABAergic populations in the cortex by 50% [8]. NKX2.1 has a critical function to induce *LHX6*, a gene required for specification and migration of MGE-derived GABAergic interneurons [12,13]. After migrating to the cortex, these LHX6^+^ neurons mature *in situ* into PV and SST cortical interneurons [11,12,14].

While significant advances have been made in understanding interneuron development [15,16], our knowledge about the intrinsic and extrinsic control of MGE progenitor proliferation, neurogenesis and specification into distinct interneuron subpopulations remains limited. Signaling by the transforming growth factor β (TGFβ) family plays a central role in multiple aspects of neural development. Through waves of activation, inhibition and reactivation, TGF-β family members exert diverse functions from patterning, neuron versus glia fate commitment, cell migration, synaptogenesis and cell survival [17]. TGFβ3 is expressed in the subventricular and mantle zones of mouse MGE and its expression is down-regulated consequent of Nkx2.1 knockout. Moreover, the *TGFβ3* gene contains both LHX6 and NKX2.1 binding sites in its enhancer [18]. Transcriptional profiling also revealed the presence of several other TGFβ members and their signaling components in the subpallium [19]. These studies implicate TGFβ3 signaling as part of a regulatory network governing MGE development.

*In vitro* differentiation of human embryonic stem cells (hESCs) or induced pluripotent stem cells (iPSCs) provides a valuable tool for studying the function of genes and signaling pathways in cell fate decisions during human neural development. Using hESC interneuron differentiation as an experimental model [20–22], we report in this study that changes in TGFβ signaling regulates human MGE progenitor cell division and transition into postmitotic neurons, hence revealing a new role for this classic signaling pathway in human interneuron differentiation.

## Materials and methods

### hESCs culture and neuronal differentiation

The hESC lines used in the present study are the H7 (also named WA07, Wicell) hESCs and two H7-derived LHX6-mCherry interneuron reporter lines, referred to as LHM12 and LHM39. The reporter lines contain a targeted insertion of mCherry in the 3′UTR of the *LHX6* gene. The two lines behave similarly and all experiments were performed with clone LHM12 at least twice and repeated at least once with LHM39. For simplicity they were referred together in short as LHM. All hPSCs were cultured on matrigel-coated plastics in E8 media (Thermo Fisher). Media were changed daily and cells passaged mechanically with 0.02% EDTA at 80% confluence. MGE differentiation followed procedures that were described previously [22]. Briefly, cells from two 80% confluent wells of a 6-well plate were plated on to a 12-well plate previously coated with reduced growth factor matrigel (VWR) in E8 media (day 0) and changed to N2B27 the next day. Cells were induced to neuroectoderm fate by LDN-193189, SB-431542 and XAV-939 from days 1 to 10 followed by ventralization with a combination of recombinant Sonic Hedgehog (SHH) and a small molecule SHH agonist purmorphamine (PM) from days 11 to 20. To promote terminal differentiation and cell survival, cultures were treated with BDNF from day 25 until analysis. Cortical differentiation followed the same procedure without XAV, SHH and PM. Where indicated MGE cultures were treated with recombinant TGFβ3 (1 ng/ml) or small molecule TGF receptor inhibitor LY2109761 (5 µM) or GW788388 (5 µM) for the time indicated. Enforcement of cell cycle exit was performed by adding DAPT (10 µM) and PD0332991 (2 µM) to differentiation cultures from days 35 to 60.

### Immunofluorescence and immunohistochemistry

Cultures were fixed with 3.7% PFA for 15–20 min at 4ºC. For nuclear antigen detection an additional fixation with methanol gradient was performed, which included 5 min each in 33 and 66% methanol at room temperature followed by 100% methanol for 20 min at −20ºC. Cultures were then returned to PBST via inverse gradient and were then permeabilized during three 10-min washes in 0.3% Triton-X-100 solution in PBS (PBS-T) and then blocked in PBS-T containing 1% BSA and 3% donkey serum. Cells were incubated with primary antibodies in blocking solution overnight at 4°C. Following three PBS-T washes, Alexa-Fluor secondary antibodies (Thermo Fisher Scientific) were added at 1:1000 in blocking solution for 1 h at ambient temperature in the dark. Three PBS-T washes were then performed that included once with DAPI at 1:1000 (Thermo Fisher Scientific). Images were taken on a Zeiss LSM710 confocal microscope from at least five randomly selected fields/sample and staining quantification was acquired manually in ImageJ (imagej.net). The antibodies used are provided in the Supplementary Table S1.

### EdU labeling and detection

Cells in S-phase were labeled using the Click-iT EdU Assay kit (Thermo Fisher). For EdU-pulse analysis, cells were incubated with EdU for 2 h at specified day of differentiation. For EdU-cumulative assay required for calculating cell cycle length, cells were incubated for increasing periods of time of 2, 4, 8, 16 and 24 h. Cells were then fixed in 3.7% PFA for 15 min at 4°C. EdU detection was carried out as per manufacturer’s protocol. Cell cycle length determination was performed using the Nowakowski equation [23]. Six fields from two biological replicates were counted at each time point of the EdU cumulative labeling. The percentages of the NKX2.1^+^EdU^+^ and NKX2.1^−^EdU^+^ in DMSO and LY conditions were plotted against time. A linear regression was performed, and the values of the slope (m) and y-intercept (b) of the linear equation were used to calculate the values of cell cycle length (tc) and S-phase length (ts), using the Nowakowski equation:

GF(t) = (GF/tc)t + GF(ts/tc). Where: GF, growth fraction in cell population; m = GF/tc, thus, tc = GF/m, b = GF(ts/tc) thus ts =(b*tc)/GF

### Flow cytometry

LHM differentiation cultures were dissociated to single cell suspension using accutase at 37°C for 5–10 min, depending on the degree of maturation. Cells were then washed with cold PBS by centrifugation and resuspended in 200 µl of cold PBS containing DAPI (1:6000). Data for mCherry^+^ cells was acquired using a Fortessa analyzer. Parental H7 cells at the same stage of differentiation were used at each time-point as negative controls. DAPI staining was used to discard dead cells from the analysis.

### Statistical analyses

All data were collected from at least three independent experiments including both LHM12 and LHM39. Unless specified data were presented as mean ± SEM. Data were tested for normality with the Shapiro–Wilk test and for equal variance with Levene test before performing statistical analyses by unpaired *t* test, ANOVA or non-parametric alternatives as stated in the figure legends where relevant. Post-hoc Tukey’s test was applied following ANOVA for multiple comparisons. All statistical tests were performed in SPSS (IBM).

## Results

### Cortical interneuron differentiation of hESCs

**Figure 1:**
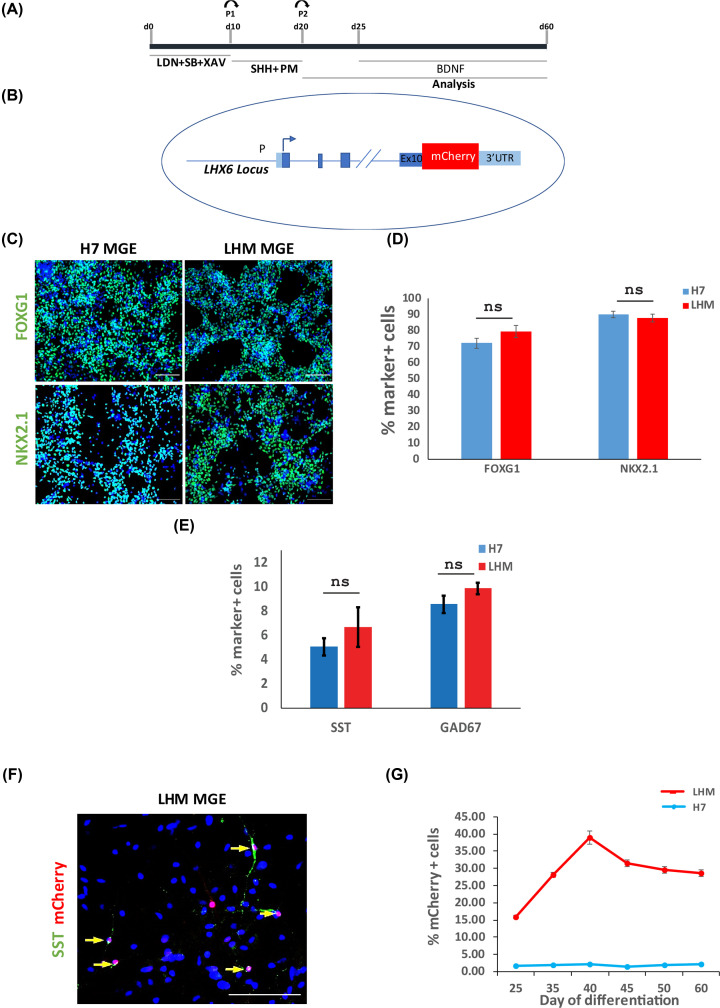
MGE induction and interneuron differentiation of hESCs (**A**) Schematic illustration of hESC interneuron differentiation protocol. (**B**) Illustration of LHX-6-mCherry knock-in hESCs. (**C**) Day 21: LHM and H7 differentiation cultures were immunostained for neural progenitor markers representing pan-forebrain (FOXG1) and MGE (NKX2.1) with DAPI counter stain (blue). (**D**) Quantification of antibody staining exemplified in (C). No statistical difference was found between the parental H7 and LHM data. *P*>0.05, two-tailed *t* test. (**E**). Quantification of the SST and GAD67 neurons in LHM and H7 cultures at day 65 of differentiation. *P*>0.05, two-tailed *t* test. (**F**). Antibody staining of day 65 cultures showing co-expression of mCherry with STT (yellow arrows), DAPI counter stain. (**G**) Flow cytometry quantification of mCherry^+^ cells during 60-day differentiation of LHM and H7 control cells with demecolcine. Scale bars 100 μm in (C,F).

To generate MGE-derived cortical interneurons, hESCs are induced toward forebrain neural progenitors by dual-Smad inhibition, followed by ventral patterning of these progenitors by SHH and PM [20–22]. Interneuron differentiation is a lengthy process, typically the expression of defined interneuron subtype protein markers such as SST will not be detected until day 60 of the protocol. To facilitate the monitoring of interneuron birth, we took advantage of an LHX6-based interneuron reporter hESC line (the LHM cells) which harbors a targeted mCherry reporter in the *LHX6* gene, which begins its expression in immature migrating cortical interneurons (Figure 1A,B) [13,24]. We firstly compared interneuron differentiation propensity of the LHM cells to the H7 parental hESCs. At day 21, the majority of hESC-derived neural progenitors derived from both hESC lines acquired MGE characteristics as demonstrated by the expression of NKX2.1 and the pan forebrain marker FOXG1 (Figure 1C,D, Supplementary Figure S1). There were similar numbers of NKX2.1^+^, FOXG1^+^ cells in LHM and H7 cultures. Moreover, at day 65 when a significant proportion of cells became postmitotic neurons, we detected a similar number of cells positive for SST and pan GABAergic marker GAD67 and that mCherry was co-expressed with SST in these cultures (Figure 1E,F). These findings indicate that the LHM reporter line has similar interneuron differentiation capacity to H7.

We next examined the temporal progression of mCherry^+^ cells during a 60-day differentiation time window by flow cytometry. No mCherry^+^ cells were detected in the H7 parental control cultures at any time points examined. At day 25, approximately 15% of cells in the LHM cultures were mCherry^+^, it rose to approx. 40% by day 40 and the level remained relatively stable thereafter (Figure 1G). We therefore chose days 25–45 as the time window for subsequent flow cytometry analysis.

### TGFβ3 accelerates immature interneuron production during hESCs differentiation

**Figure 2:**
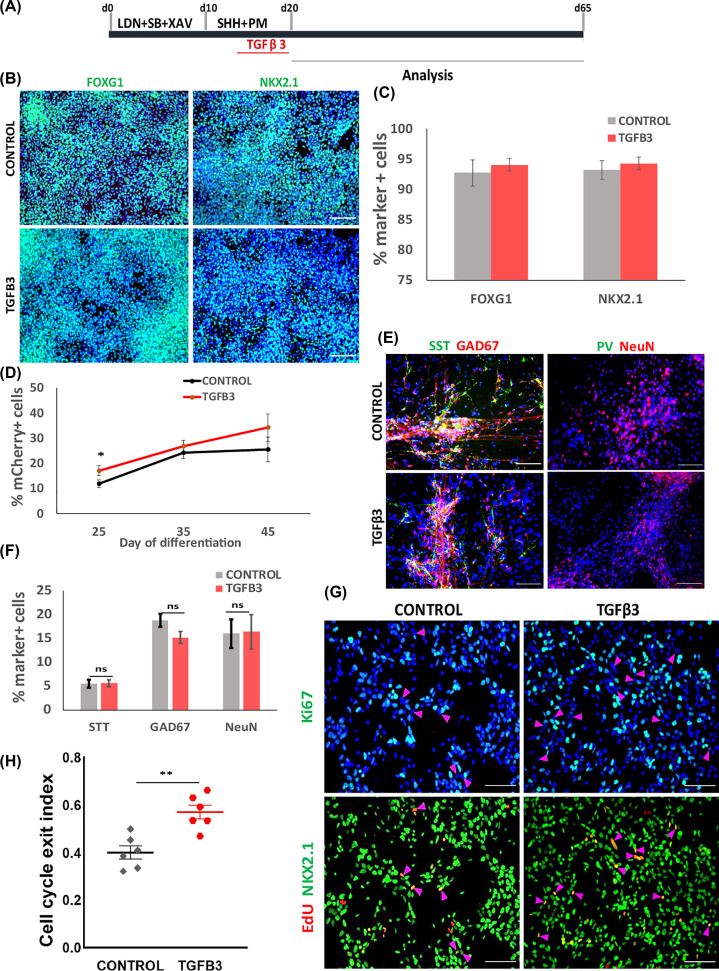
Effects of exogenous TGFβ3 on hESC MGE differentiation (**A**) Illustration of TGFβ3 experimental design. (**B**) Day 21 control and TGFβ3 treated cultures of LHM12 and H7 cells were immunostained for FOXG1 and NKX2.1 with DAPI counter stain (blue). (**C**) Quantitative data of NKX2.1^+^ and FOXG1^+^ cells. Data represent mean ± SEM of two independent differentiation each with duplicate wells. No significant difference was found between the control and TGFβ3 conditions, *P*>0.05, two-tailed *t* test. (**D**) Effect of TGFβ3 on mCherry^+^ cell production measured by flow cytometry. Significant difference was found at day 25, * P<0.05, two-tailed *t* test. (**E**) Immunocytochemistry for NeuN, GAD67, SST and PV at day 65 with DAPI counterstain. (**F**) Quantification of STT^+^, GAD67^+^, NeuN^+^ cells. *P*>0.05, two-tailed *t* test. (**G**) Triple antibody staining of LHM12 and LHM39 cultures for EdU, Ki67 and NKX2.1 with DAPI counterstain. The two images on the top and bottom, respectively, are from the same field of view, the magenta arrows indicate cells leaving the cell cycle. (**H**) Quantification of cell cycle exit index (ratio of NKX2.1^+^EdU^+^Ki67^−^ cells to the total EdU^+^ population). Data are presented as the median percentage with the first and third quartiles from two independent differentiation. **P<0.01, two-tailed *t* test. Scale bar in (B,E,G): 100 μm.

TGFβ3 and its downstream signaling molecules are expressed in the developing MGE while TGFβ3 enhancer region also contains Nkx2.1 and Lhx6 binding sites [18,19]. These findings present TGFβ signaling a strong candidate player in interneuron development. Both TGFβ3 and TGFβ receptor transcripts are expressed during interneuron differentiation of hESCs (Supplementary Figure S2). To investigate whether exogenous TGFβ3 could elicit any effect, recombinant TGFβ3 was added during the ventrolization stage of days 14–20 followed by immunostaining for FOXG1 and NKX2.1 at day 21 (Figure 2A). No significant difference in the number of FOXG1^+^ and NKX2.1^+^ cells were found between TGFβ3 treated and control cultures (FOXG1: 94.06 ± 1.06 vs 92.71 ± 2.17%; NKX2.1: 94.28 ± 2.11 vs 93.23 ± 1.56%; Figure 2B,C), suggesting that exposure to TGFβ3 did not affect MGE progenitor production. We then performed flow cytometry analysis to detect mCherry^+^ cells at days 25, 35 and 45 of differentiation. We found that TGFβ3 treated cultures tend to contain a higher number of mCherry^+^ cells than the controls, although statistical significance was only confirmed for day 25 (Figure 2D). Intriguingly, when examined at day 65 when the cultures were more mature and interneuron subtype marker expression began to appear, we found no difference in the proportion of SST^+^, GAD67^+^ or NeuN^+^ neurons between TGFβ3-treated and control cultures (Figure 2E,F, Supplementary Figure S3).

Together the above observations suggest that TGFβ3 may accelerate postmitotic differentiation of MGE progenitors. To test this possibility we exposed day 21 MGE progenitors to a 2-h EdU pulse followed by triple immunostaining staining for EdU, Ki67 and NKX2.1 at day 23. This experiment allowed the determination of the cell cycle exit index which is the ratio of EdU-labeled progenitors already left the cell cycle (NKX2.1^+^EdU^+^Ki67^−^) to the total numbers of EdU^+^ cells. Indeed, TGFβ3 treatment led to an increase in cell cycle exit index compared with the controls (Figure 2G,H).

### TGFβ signaling inhibition delays the production of interneuron

**Figure 3:**
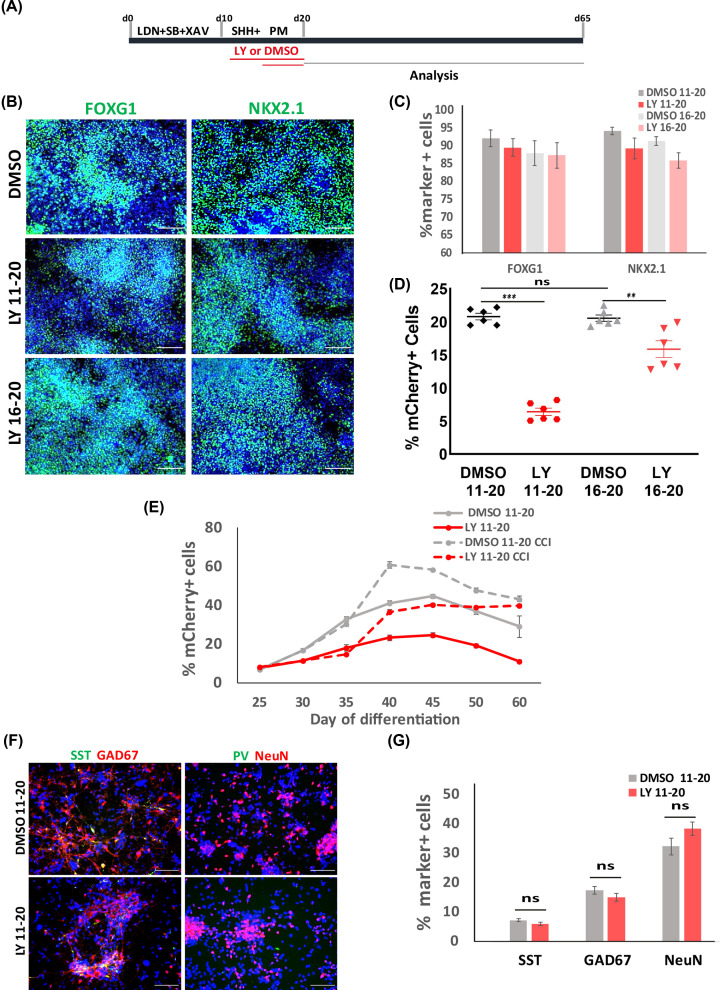
Inhibition of TGFβ signaling favors MGE progenitor maintenance (**A**) Illustration of TGFβ inhibition experiments. (**B**) Immunostaining for MGE markers at day 21 with DAPI counterstain. (**C**) Quantitative analysis of MGE marker^+^ cells. (**D**) Flow cytometry measurement of mCherry^+^ cells at day 35. ns, *P*>0.05, ** P<0.01, ***P<0.001, one-way ANOVA with post-hoc Tukey’s test. (**E**) Flow cytometry measurement of mCherry^+^ cells (LHM12 and LHM39) during 60-day differentiation under conditions indicated. Data are presented as mean ± SEM of three independent sets of experiments. (**F**) Immunostaining for key interneuron markers with DAPI counter stain. (**G**) Quantification of (F). *P*>0.05, two-tailed *t* test. Scale bar in (B,F): 100 μm.

**Figure 4:**
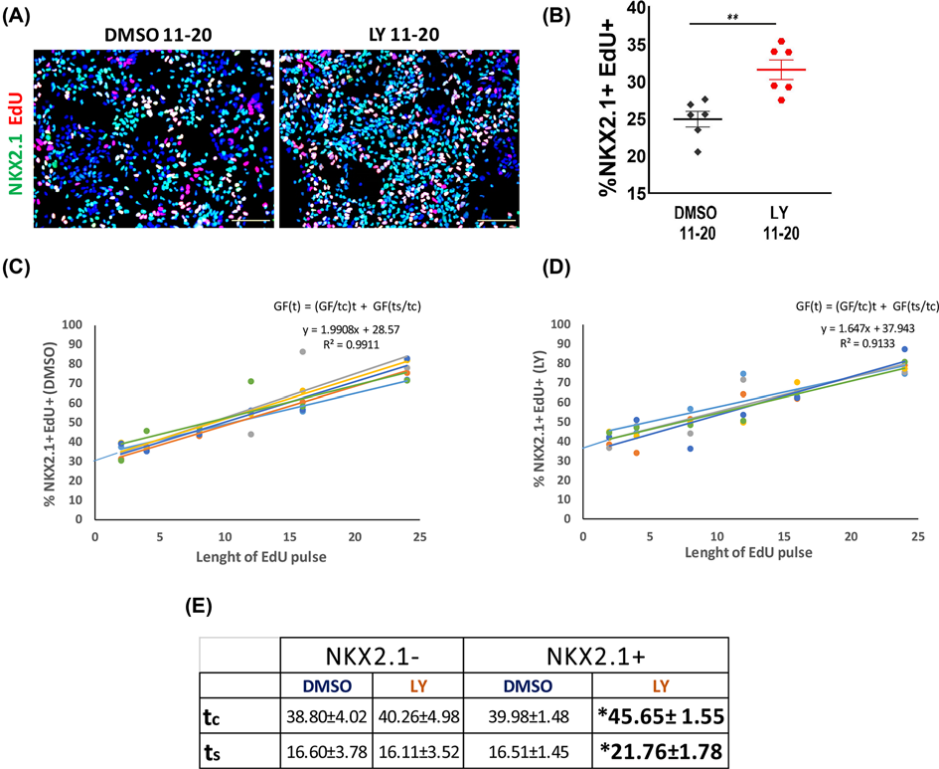
TGFβ signalling regulates cell cycle length (**A**) Double staining for NKX2.1 and EdU with DAPI counterstain in day 22 control and LY cultures of LH12 and H7 cells. (**B**) Quantification of H, **P<0.01, two-tailed *t* test. (**C**,**D**) Representative plots of EdU incorporation within NKX2.1^+^ population of LHM12 and LHM39 cells in DMSO and LY conditions, respectively. Nowakowski equation used for calculating cell cycle length. (**E**) Table summarizing the tc and ts of NKX2.1^+^ and NKX2.1^−^ population with or without LY treatment. Data are presented as average length (hours) of tc and ts ± SEM from two independent experiments with three technical replicates. For NKX2.1^+^ population tc: *P=0.025, two-tailed *t* test, equal variances assumed, ts: *P=0.045, two-tailed *t* test. For NKX2.1^−^ population, no significant differences were found, tc: *P*=0.832, two-tailed *t* test, equal variances assumed, ts: *P*=0.927, two-tailed *t* test. Scale bar in (A): 100 μm.

To further elucidate the role of TGFβ signaling in interneuron differentiation, we performed pharmacological inhibition of this pathway using a selective inhibitor of type I/II TGFβ receptor LY2109761 (referred to as LY thereafter). The LHM and parental H7 cells were treated with DMSO vehicle or LY between days 11–20 and days 16–20 of interneuron differentiation (Figure 3A). At day 21, a similar proportion of FOXG1^+^ and NKX2.1^+^ cells were found in LY- and DMSO-treated cultures in both cell lines (Figure 3B,C, Supplementary Figure S4). However, a snapshot flow cytometry analysis of day 35 cultures revealed a four-fold decrease in mCherry^+^ cells in LY days 11–20 treated LHM cultures compared with DMSO controls, while the LY days 16–20 resulted in a 25% decrease (Figure 3D). We therefore examined more closely the effects of LY treatment on mCherry^+^ cell production over a 60-day culture period using the LY days 11–20 treatment regime (Figure 3D). At day 25, no difference was found in the proportion of mCherry^+^ cells between the LY-treated and control cultures (Control: 7.1 ± 0.23%, LY: 7.58 ± 0.40%, Figure 3E, solid lines). However, from day 30 onwards LY cultures contained less mCherry^+^ cells compared with the controls, reaching a maximum 45% reduction at day 45 (control: 44.72 ± 1.06%, LY: 24.62 ± 1.11%, Figure 4E). A decrease in mCherry^+^ cells was also observed with the use of another TGFβRI inhibitor GW788388 (Supplementary Figure S5). Intriguingly, at day 65 the LY and control cultures contained a similar proportion of GAD67^+^ and SST^+^ cells while no PV^+^ cells were detected in either conditions (Figure 3F,G, Supplementary Figure S3).

### TGFβ signaling regulates cell cycle length

The transient decrease in interneuron production suggests that low TGFβ signaling environment may favor neural progenitor maintenance. To test this hypothesis we first measured EdU incorporation rate of NKX2.1^+^ progenitors after a 2-h pulse at day 22. Double staining for NKX2.1 and EdU revealed a significant higher proportion of EdU^+^NKX2.1^+^ cells in the LY cultures than those in controls (Figure 4A,B). Consistent with this observation, RT-PCR analysis spanning 60-day differentiation showed that transcripts associated with proliferating MGE progenitors (*TOP2A*, *FABP7, TACC3* and *CENPF)* were detected at a higher level in a time-dependent fashion in LY-treated cultures than that in control cultures [16] (Supplementary Figure S6). Conversely, the level of postmitotic neuron-associated transcripts (*STMN2, GAD1, SOX6* and *LHX6*) were higher in controls than LY-treated cultures (Supplementary Figure S6).

The above findings are intriguing and seemingly contradictive to the observed lack of increase in NKX2.1^+^ cells (Figure 3B,C). We therefore investigated a potential change in cell cycle kinetics. At day 22, LY-treated and control cells were exposed to cumulative EdU from 2 to 24 h followed by EdU and NKX2.1 double staining. The length of cell cycle (tc) and S-phase (ts) were then calculated using the method of Nowakowski equation (Figure 4C,D) [23]. LY treatment did not affect the tc (DMSO: 38.80 ± 4.02 h, LY: 40.26 ± 4.98 h) or ts (DMSO: 16.60 ± 3.78 h, LY: 16.11 ± 3.52 h) in NKX2.1^−^ population (Figure 4E). However, NKX2.1^+^ cells treated with LY had a concomitant increase in tc and ts compared with DMSO controls (tc/DMSO: 39.98 ± 1.48 h, tc/LY: 45.65 ± 1.55 h; ts/DMSO: 16.51 ± 1.45 h, ts/LY: 21.76 ± 1.78 h, Figure 4C–E).

In the developing mouse telencephalon, a long ts is associated with neural progenitors committed to proliferation rather than those undergoing neurogenic division [25]. To gain further support that LY treatment promotes the maintenance of cycling progenitors, we exposed the differentiation cultures to two cell cycle inhibitors (CCIs): γ-secretase inhibitor DAPT and CDK4/6 inhibitor PD0332991, with or without LY from day 35 to 60, and examined the numbers of mCherry^+^ from day 40 to 60 by flow cytometry (Figure 3E). Addition of CCI resulted in an increase in mCherry^+^ cells in both the basal control (no LY) and LY-treated cultures (Figure 3E, dashed lines). Interestingly, following CCI treatment, the number of mCherry^+^ cells in LY-treated cultures rose to the level of basal control cultures at day 40 and even reach the same level to CCI-treated control cultures at day 60 (Figure 3E, dashed lines).

Together, these findings support the notion that low TGFβ signaling bias the maintenance of progenitor state, resulting in a protracted terminal differentiation as reflected in the temporal reduction in postmitotic LHX6^+^ neurons. However, progenitors experienced LY remain competent in neurogenesis and are able to undergo terminal differentiation when LY action eventually fades.

## Discussion

The present study identifies a new role for TGFβ signaling in cortical interneuron neurogenesis. Currently, generation of cortical interneurons from hPSCs remains a challenge. Although induction of NKX2.1^+^ MGE progenitors can be achieved reliably and highly efficiently [20–22,26,27], only a proportion of these progenitors progress to LHX6^+^ postmitotic interneurons. Moreover, the yield of defined interneuron subtypes (SST^+^ or PV^+^) remains very low [20–22,26] or the phenotype is unstable [28]. This is largely due to our limited understanding of the molecular machinery controlling interneuron diversity and subtype specification, which is further complicated by the protracted acquisition of interneuron subtype identity both during normal development and hPSC differentiation [21,29,30].

A role for TGFβ signaling in regulating progenitor proliferation versus terminal neuronal differentiation has been reported previously in the developing dorsal-caudal midbrain and the cortex [31,32]. TGFβ works in concert with Wnt signaling for its action in the midbrain [31]. Inhibition of TGFβ signaling in this region by *Tgfbr2* ablation led to ectopic expression of Wnt1 and increased accumulation of β-catenin, which in turn activates Cyclin D1 transcription in dorsal midbrain progenitors, thereby promoting their proliferation. Interestingly, activation of WNT/β-catenin signaling was recently shown to promote the maintenance of MGE-like progenitors derived from hPSCs, while ablation of *CTNNB1* led to early cell cycle exit and prompts neurogenesis of these progenitors [33]. In addition to cell cycle regulation, WNT signaling has also been shown to play a role in interneuron subtype specification through Ryk receptor, which activates non-canonical Wnt signaling [34]. Whether our finding of TGFβ-regulation on MGE progenitor neurogenesis involves interaction with WNT signaling remains an interesting future prospective.

In addition to controlling proliferation and neurogenesis, studies on *Tgfβr1*-deficient mice suggest a role for TGFβ signaling in neuronal maturation, as these mice display a decrease in neuronal number as well as their migration capacity and dendritic arborization in the adult hippocampus [35]. Independent studies show that enforced cell cycle exit of GABAergic progenitors accelerate neuron maturation at electrophysiological level [36]. These studies raise an interesting question whether TGFβ3 may also promote cortical interneuron maturation and be exploited for producing these disease relevant neuronal types from hPSCs.

In summary, the current study uncovered a new function of TGFβ signaling in human interneuron development. During development, MGE progenitors exhibit a temporal bias in giving rise to PV or SST interneuron cell types, therefore TGFβ regulation of MGE progenitor cell division and neurogenesis could potentially offer a mean to augment interneuron subtype-specific differentiation from hPSCs.

## Supplementary Material

Supplementary Figures S1-S6 and Table S1

## Data Availability

All data generated or analyzed during the present study are included in this published article (and its supplementary information files).

## Open Access

Open access for this article was enabled by the participation of Cardiff University in an all-inclusive *Read & Publish* pilot with Portland Press and the Biochemical Society under a transformative agreement with JISC.

## Abbreviations

CCI: cell cycle inhibitor
GABA: γ-amino butyric acid
hESC: human embryonic stem cell
MGE: medial ganglionic eminence
PBS-T: 0.3% Triton-X-100 solution in PBS
PM: purmorphamine
PV: parvalbumin
SHH: Sonic Hedgehog
SST: somatostatin
TGFβ: transforming growth factor β
