# Supplementary material for: Identification of TGFβ signaling as a regulator of interneuron neurogenesis in a human pluripotent stem cell model
Source: Neuronal Signal. 2021 Dec 7;5(4):NS20210020. doi: 10.1042/NS20210020 (PMC8661503; doi:10.1042/NS20210020)
Supplement: Supplementary Figures S1-S6 and Table S1 [file NS-2021-0020_supp.pdf]

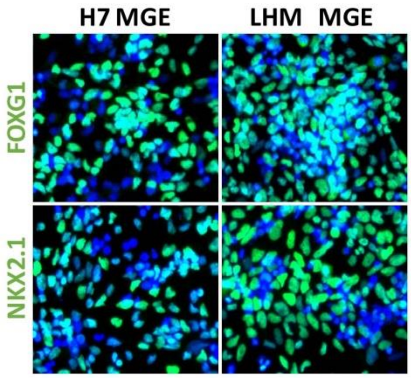


**Fig S1. MGE induction of hESCs**

Day 21 LHM differentiation cultures were immunostained for neural progenitor markers representing pan-forebrain (FOXG1) and MGE (NKX2.1) with DAPI counter stain (Blue). The panels shown are higher magnification of figure 1C.

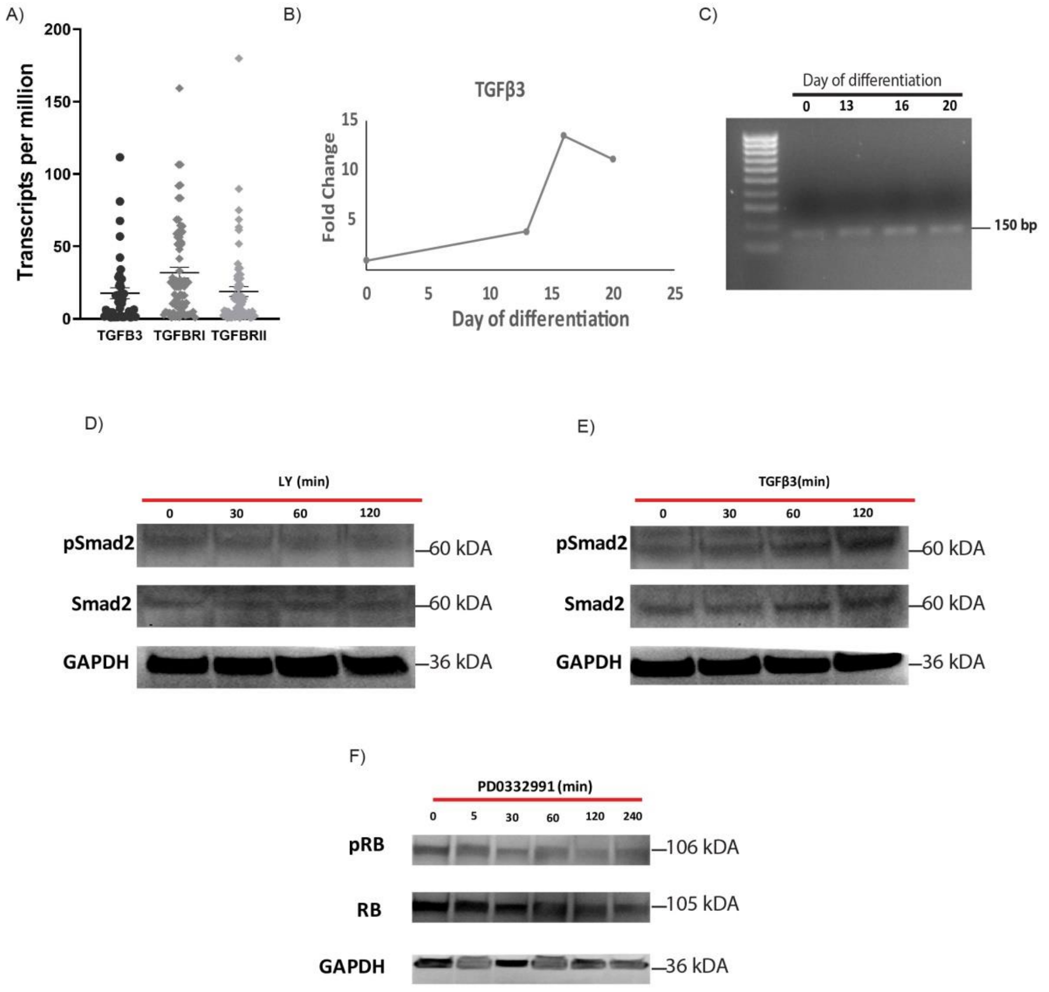


**Figure S2. Validation of biological activity of TGFβ3, TGFβ inhibitor LY2109761 and CDK4/6 inhibitor PD0332991**

A, Single cell RNAseq data showing the transcript levels of TGFβ3, TGFβRI and TGFβRII in a single hESC-derived MGE cells. n=42, 70 and 67, respectively; TPM: transcript per million.

B, qPCR analysis for TGFβ3 at day 13, 16 and 20 and shows its upregulation relative to undifferentiated hESCs at day 0;

C, Agarose gel electrophoresis showing the final 150bp qPCR product;

D, hESC-derived MGE cells were exposed to LY2109761 for the time indicated and processed for Western blot analysis for total Smad2 and phospho-Smad2;

E, Western blot analysis of TGFβ3 treated cells for total and phospho-Smad;.

F, Western blot analysis for total and phosphorylated RB protein on cells treated with the CDK4/6 inhibitor PD0332991.

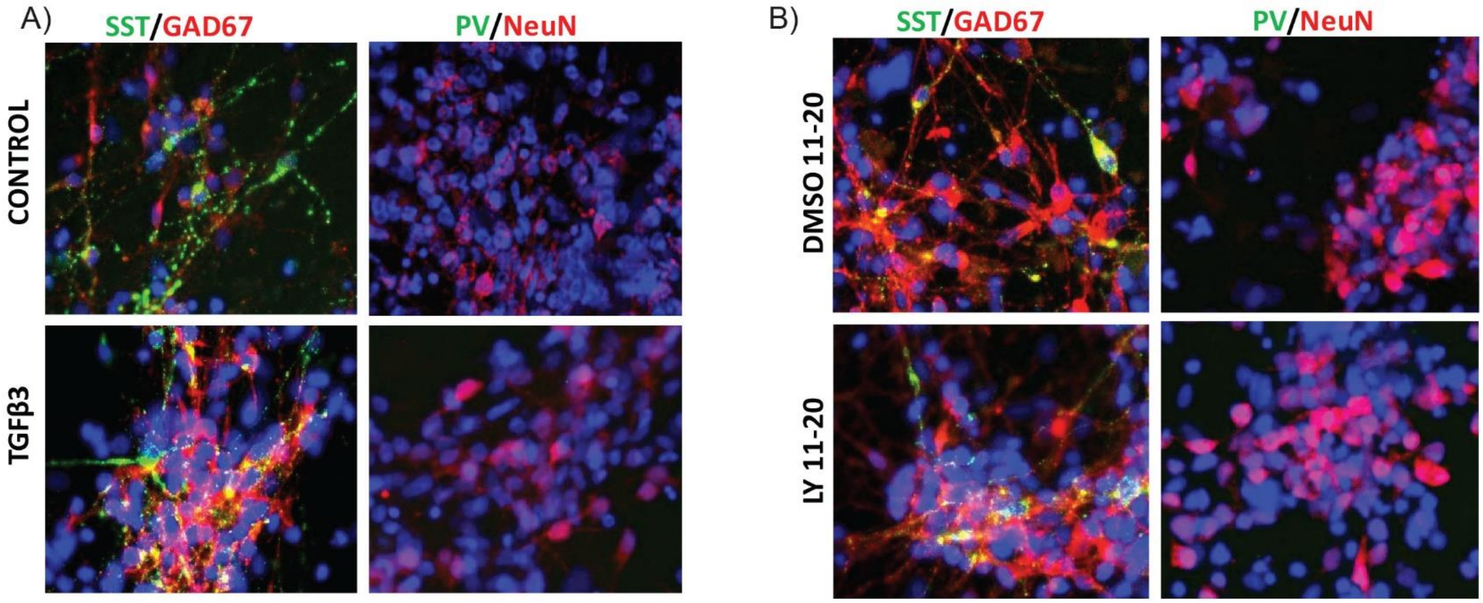


**Figure S3, Higher magnification images**
A, Digital magnification of Figure 2E,
B, Digital magnification of Figure 3F.

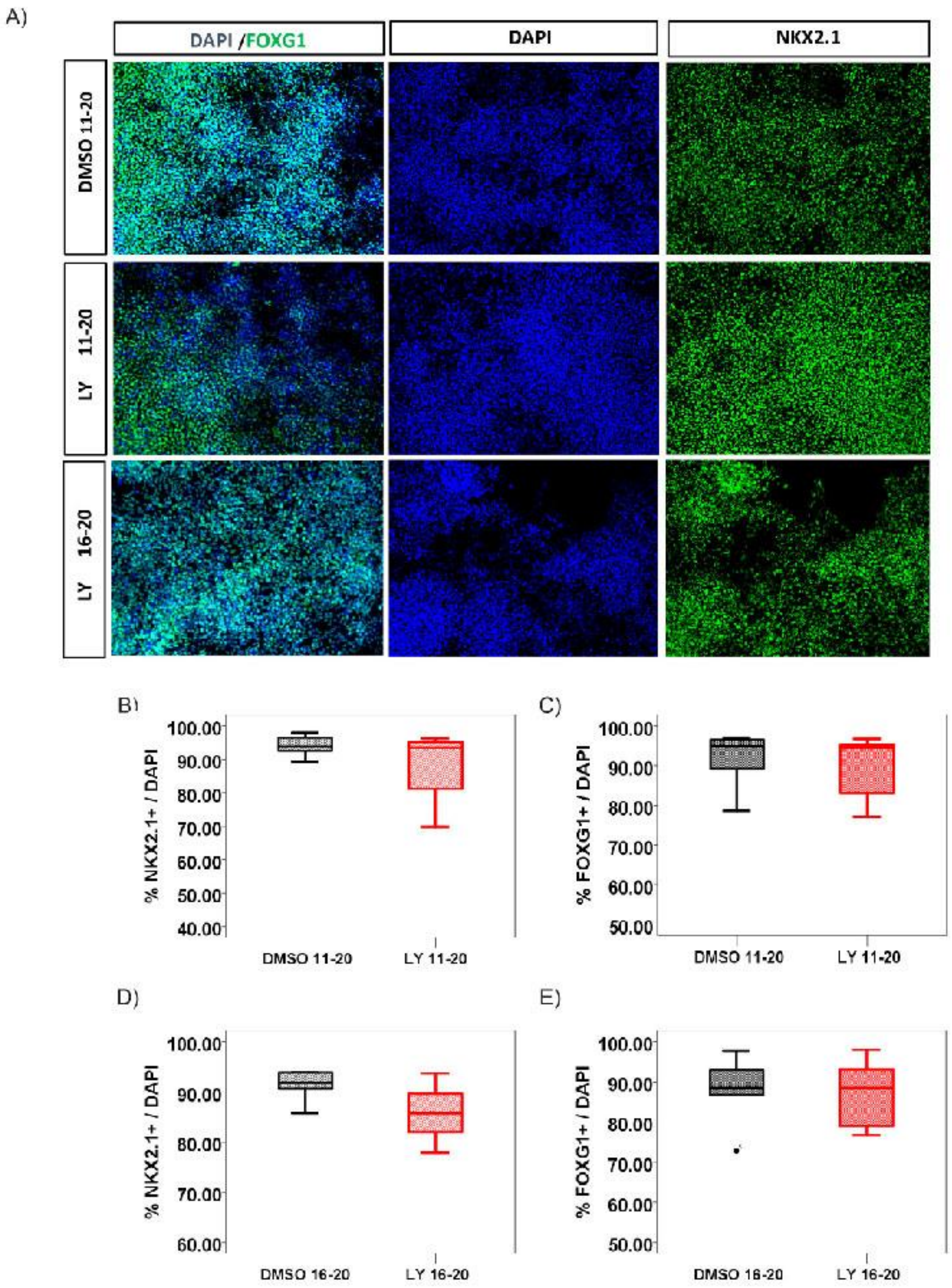
A)
DAPI /FOXG1
DAPI
NKX2.1
DMSO 11-20
LY 11-20
LY 16-20
B)
% NKX2.1+ / DAPI
DMSO 11-20
LY 11-20
C)
% FOXG1+ / DAPI
DMSO 11-20
LY 11-20
D)
% NKX2.1+ / DAPI
DMSO 16-20
LY 16-20
E)
% FOXG1+ / DAPI
DMSO 16-20
LY 16-20

**Fig S4, TGFβ inhibition doesn’t affect MGE-progenitor production**

A, Representative immunocytochemistry for NKX2.1 and FOXG1 in H7 parental line. B-E, Quantitative data of the marker-expressing cells in LY treated conditions and respective DMSO controls.

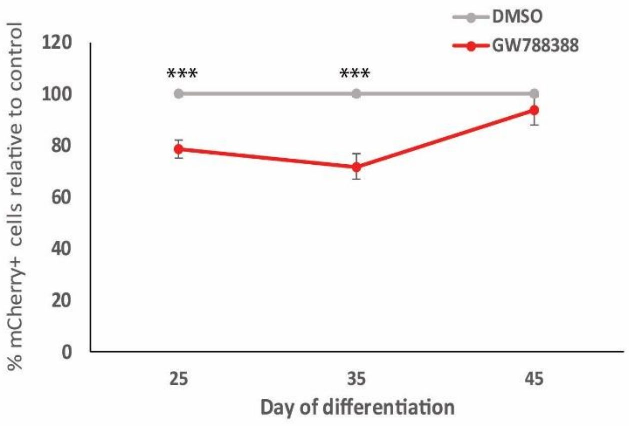


**Figure S5. Effects of TGFβ inhibitor GW788388 on mCherry$^{+}$ cell production**
Day 25 and day 35 flow cytometry profile of mCherry$^{+}$ cells of cultures treated with or without GW788388. Shown are GW788388 relative to control values where the lateral was set as 100%. *** P<0.001, univariate analysis post-hoc Bonferroni.

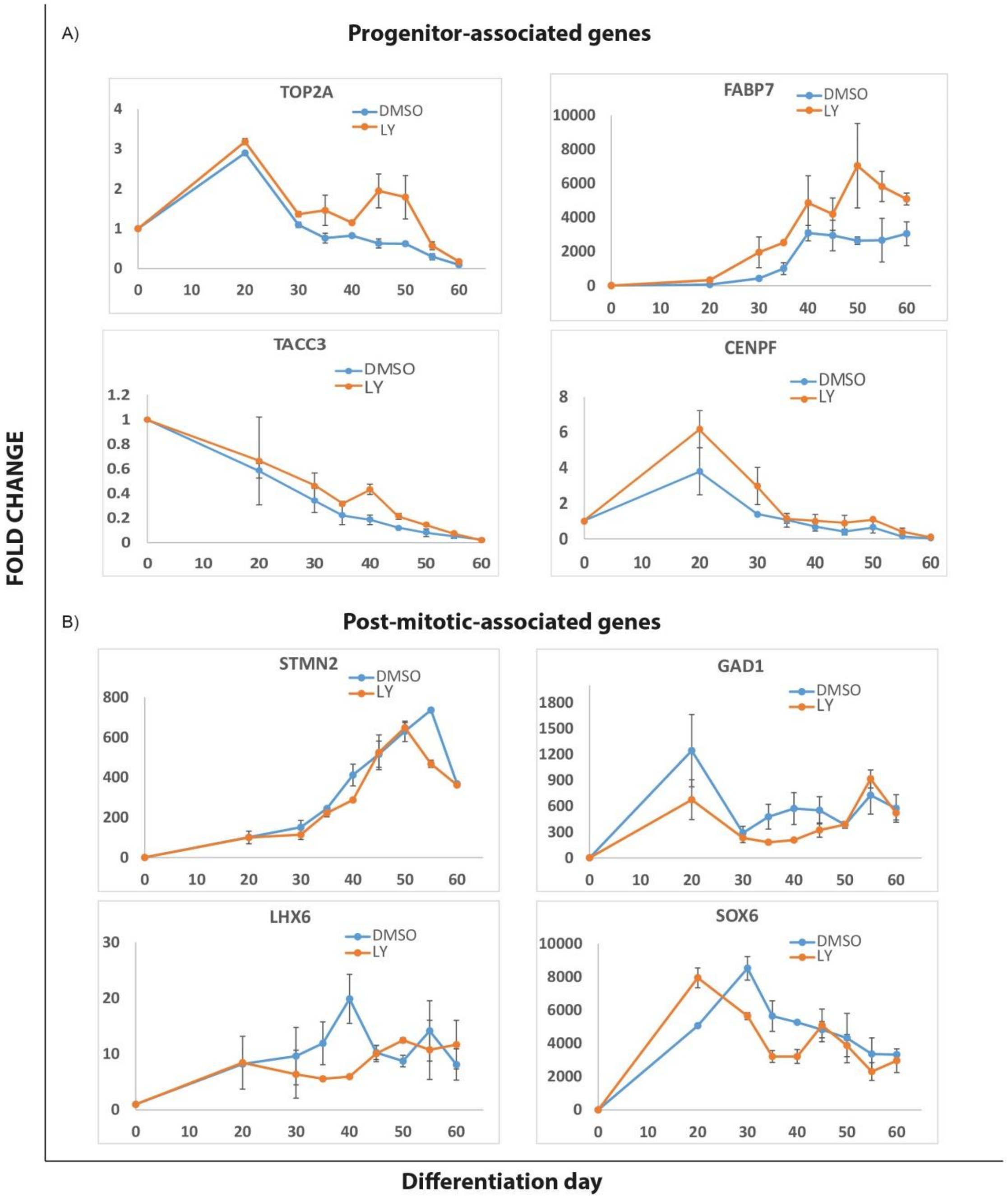

A)
Progenitor-associated genes
TOP2A
FABP7
TACC3
CENPF
DMSO
LY
B)
Post-mitotic-associated genes
STMN2
GAD1
LHX6
SOX6
FOLD CHANGE
Differentiation day

**Fig S6, Effect of TGFβ inhibition on expression of genes associated with MGE progenitors and post-mitotic neurons.**

A) Expression profile in LHM cells of MGE-progenitors associated genes (*TOP2, FABP7, TACC3* and *CENPF*) in control (blue) and LY-treated cultures (orange). B) Expression profile in LHM cells of genes associated to post-mitotic MGE derivatives (*STMN2, GAD1, LHX6* and *SOX6*) in control (blue) and LY-treated cultures (orange). Data is presented as the mean fold change ± SEM from triplicates of two independent experiments.

**Table S1** Antibodies used for immunocytochemistry

| Antigen | Species | Supplier | Code | Dilution |
|---|---|---|---|---|
| CALRETININ | Rabbit | Swant | CR 7697 | 1:500 |
| CTIP2 | Rat | Abcam | Ab18465 | 1:500 |
| FOXG1 | Rabbit | Abcam | ab18259 | 1:250 |
| GAD67 | Mouse | Millipore | mab5406 | 1:500 |
| LHX6 | Rabbit | Santa cruz | sc98607 | 1:500 |
| mCherry | CHICKEN | ABCAM | ab205402 | 1:500 |
| NeuN | RABBIT | MILLIPORE | | 1:500 |
| NKX2.1 | Rabbit | Abcam | Ab76013 | 1:1000 |
| OCT4 | Goat | Santa cruz | Sc8628 | 1:500 |
| OLIG2 | Goat | R&D systems | AF2418 | 1:200 |
| PAX6 | Mouse | DSHB | | 1:1000 |
| PARVALBUMIN | Mouse | Sigma | P3088 | 1:100 |
| SOMATOSTATIN | Rat | Millipore | MAB354 | 1:50 |
| TBR1 | Rabbit | Abcam | ab31940 | 1:500 |
